# The Characteristics and Patterns of Drug-Resistant Pulmonary Tuberculosis in Eastern India

**DOI:** 10.3390/tropicalmed7090244

**Published:** 2022-09-13

**Authors:** Vishal Prakash Giri, Om Prakash Giri, Pooja Tripathi Pandey, Kripa Nath Mishra, Ram Shanker Prasad, Prabhat Kumar Lal, Rana Pratap, Nishant Nikhil, Abu Sufian, Reyaz Ahmad, Shubhra Kanodia

**Affiliations:** 1 Department of Pharmacology, Autonomous State Medical College, Shahjahanpur 242001, India; 2 Department of Respiratory Medicine and Tuberculosis, Darbhanga Medical College and Hospital, Darbhanga 846003, India; 3 Department of Physiology, Autonomous State Medical College, Shahjahanpur 242001, India; 4 Department of Pediatrics, Darbhanga Medical College and Hospital, Darbhanga 846003, India; 5 Department of Microbiology, Darbhanga Medical College and Hospital, Darbhanga 846003, India; 6 Department of Community Medicine, Darbhanga Medical College and Hospital, Darbhanga 846003, India; 7 Department of Microbiology, Autonomous State Medical College, Shahjahanpur 242001, India; 8 Nodal Drug Resistant Tuberculosis Centre, Department of Respiratory Medicine and Tuberculosis, Darbhanga Medical College and Hospital, Darbhanga 846003, India; 9 Department of Dentistry, Autonomous State Medical College, Shahjahanpur 242001, India

**Keywords:** drug resistance, drug-resistant tuberculosis, extensively drug-resistant tuberculosis, national tuberculosis elimination program, multi-drug-resistant tuberculosis, treatment outcome, adverse drug events

## Abstract

Background: Drug-resistant tuberculosis is a major public health problem throughout the world and accounts for substantial morbidity and mortality rates in India, too. Early diagnosis is the corner stone of tuberculosis treatment. State-level and cluster-wise variations in drug resistance is a possibility and should be regularly checked in from time to time. Materials and Methods: The present prospective cohort study (January 2019 to May 2022) was conducted in Darbhanga Medical College and Hospital on drug-resistant pulmonary tuberculosis patients. Sputum specimens were collected from designated centers. Rapid molecular drug-resistance testing (genotypic tests) and growth-based drug-susceptibility testing (DST) (phenotypic tests) were performed in the National Tuberculosis Elimination Program certified Laboratory. Results: A total of 268 patients with drug-resistant pulmonary tuberculosis were included in the study group. The treatment outcomes revealed as cured in 100 (37.31%); treatment completed in 43 (16.04%); died in 56 (20.89%); treatment failed in 22 (8.21%); loss of follow up in 34 (12.69%); and transferred out in 13 (4.85%) drug-resistant pulmonary tuberculosis patients. Adverse events were recorded in 199 (74.25%) of the drug-resistant pulmonary tuberculosis patients. Conclusions: Drug-resistant pulmonary tuberculosis patients are a matter of concern and need to be addressed.

## 1. Introduction

There are thirty high multi-drug/rifampicin-resistant tuberculosis (MDR/RR-TB)- burden countries in the world. The new countries that have joined the list are Mongolia, Nepal; and Zambia and transitioned out of the list are Ethiopia, Kenya; and Thailand. Eight countries (India, Pakistan, China, Bangladesh, Philippines, Indonesia, South Africa, and Nigeria) accounted for two-thirds of global cases in 2020. The World Health Organization’s End-TB strategy envisions a world free of TB (zero deaths, disease, and suffering due to TB); the goal is to end the TB pandemic and the targets are a ninety-percent reduction in the absolute number of TB deaths, an eighty-percent reduction in the TB incidence rate (compared to the 2015 baseline), and a zero-percent rate of TB-affected households facing catastrophic costs due to TB (level in 2015 unknown) by 2030. The coronavirus disease (COVID-19) has had a severe impact on TB-detection and mortality rates. An estimated 1.4 million fewer people received TB-care services in 2020 than in 2019 [1,2,3].

In India, the National Tuberculosis Elimination Program (NTEP) has developed a National Strategic Plan (NSP) 2017–2025 to achieve the milestone of eliminating TB from the country by 2025, and to achieve this target, Universal Drug Susceptibility Testing (UDST) and bedaquiline containing treatment regimens have been rolled out across the country. NTEP has established an efficient system of the targeted delivery of patient-support benefits. NIKSHAY (the word is a combination of two Hindi words; Ni = end, kshay = TB), an online tool for monitoring the TB-control program, has been launched and the Public Fund Management System (PFMS) has been established to provide direct-benefit transfer [4]. The WHO has approved molecular assays Xpert *MTB*/RIF (CBNAAT), Xpert Ultra, and Truenat as initial tests for the diagnosis of tuberculosis (TB) and rifampicin resistance [3].

Drug-resistant-tuberculosis (DR-TB) patients are classified as rifampicin-resistant TB (RR-TB), multidrug-resistant TB (MDR-TB), and extensively-drug-resistant-TB (XDR-TB) patients. The different regimens used for DR-TB treatment are the shorter oral bedaquiline-containing MDR/RR-TB; longer oral M/XDR-TB; and isoniazid (H) mono/poly DR-TB.

A shorter oral bedaquiline-containing regimen consists of an initial phase (IP) of four months that may be extended to six months, and a continuation phase (CP) of five months; thus, the total duration of the regimen is nine to eleven months. Bedaquiline is used for six months. From the start to the end of the fourth month—bedaquiline (Bdq), levofloxacin (Lfx), clofazimine (Cfz), pyrazinamide (Z), ethambutol (E), high-dose isoniazid (H), and ethionamide (Eto) are used; from the start of the fifth to the end of the sixth month—(if IP is not extended)—Bdq, Lfx, Cfz, Z, and E are used; and from the seventh to the end of the ninth month—Lfx, Cfz, Z, and E are used. The longer oral M/XDR-TB regimen lasts for a total duration of eighteen to twenty months with no separate initial or continuation phases, and the drugs used are levofloxacin (Lfx), bedaquiline (6 months or longer), linezolid (Lzd), clofazimine (Cfz), and Cycloserine (Cs). The H-mono/poly DR-TB regimen lasts for a total duration of six to nine months with no separate initial or continuation phases, and consist of levofloxacin (Lfx), rifampicin (R), ethambutol (E), and pyrazinamide (Z) [4].

The End-TB program requires further research and development, as well as an up-to-date assessment of the TB epidemic at global, regional, and country levels. Hence, the present study was planned to address these concerns. The aims of this study are to determine the characteristics and patterns of drug-resistant pulmonary tuberculosis in North Bihar.

## 2. Materials and Methods

The present prospective cohort study (January 2019–May 2022) was conducted in the Nodal Drug Resistant Tuberculosis Centre (NDR-TBC), Darbhanga Medical College and Hospital (DMCH), Bihar, India. The study was approved by the Institutional Ethics Committee of Darbhanga Medical College. Written informed consent was obtained from each patient participating in the study.

### 2.1. Inclusion Criteria

Bacteriologically confirmed, notified, pulmonary tuberculosis cases in the Darbhanga district comprised the study group. Patients of both genders and all age groups were considered.

### 2.2. Exclusion Criteria

The patients of neighboring districts and drug-resistant extra-pulmonary tuberculosis were not included in the study group.

### 2.3. Protocol of the Study

The Damien Tuberculosis Research Centre (DTRC) Darbhanga, which is situated in the campus of the Darbhanga Medical College and Hospital, is a National TB Elimination Program (NTEP)-certified C-DST (culture and drug-susceptibility test) laboratory, and is well equipped with molecular diagnostics (cartridge-based nucleic acid amplification test and line probe assays) as well as liquid culture–drug-susceptibility testing (LC-DST).

Rapid molecular diagnostic tests used for the microbiological confirmation of TB were the cartridge-based nucleic acid amplification test (CBNAAT) (also called Xpert *MTB*/Rif test) and line probe assay (LPA).

The device used for CBNAAT was the Gene Xpert system. This system was composed of a CBNAAT cartridge, Gene Xpert instrument, and computer (desktop/laptop). Each CBNAAT cartridge contained an internal control (sample-processing control and probe control check); all elements necessary for the reaction (lyophilized reagents, liquid buffers, and wash solutions). The specimen was inoculated in the CBNAAT cartridge and then the CBNAAT cartridge was loaded into the Gene Xpert instrument. Each Gene Xpert instrument of the DTRC was linked to a computer (laptop) with a printer for data analysis. The result of the CBNAAT was available in a printed format as (a) *MTB*—detected/not detected and (b) rifampicin resistance—detected/not detected/indeterminate. Laboratory turnaround time (time taken from the receipt of a sputum specimen at DTRC to the issue of the result) was two hours.

Rifampicin resistance detected (by CBNAAT) sputum specimens was subjected to a first line-line probe assay (FL-LPA), second line-line probe assay (SL-LPA), and liquid culture-drug susceptibility testing (LC-DST) by microbiologists from the C-DST laboratory (DTRC, Darbhanga). Rifampicin resistance not detected by (CBNAAT) sputum specimens was subjected to FL-LPA, and if H (isoniazid) resistance was detected by FL-LPA, then the sputum specimens were subjected to SL-LPA and liquid culture drug-susceptibility testing.

A mycobacterial growth-indicator tube liquid culture system (BACTEC MGIT 960 System) is an automated liquid culture system for liquid culture and the phenotypic drug-susceptibility testing of sputum specimens to many anti-TB drugs. This system consists of 7 mL round-bottom plastic tubes (MGIT tubes). Each MGIT tube contains modified Middlebrook 7H9 broth base and has an oxygen-sensitive fluorescent sensor at the bottom of the tube. When *mycobacteria* grow, they deplete the dissolved oxygen in the broth and allow the sensor to brightly fluorescence. The MGIT system automatically detects this fluorescence. The detection of fluorescence by the MGIT system indicates the growth of *mycobacteria*. The laboratory turnaround time for the culture on an MGIT system is 8–10 days for smear-positive specimens, and 2–6 weeks for smear-negative samples. The MGIT SYSTEM is also used for DST. If a drug is active against *mycobacteria*, it inhibits the growth and suppresses the fluorescence. The time to result for DST is 15–28 (maximum 42) days.

### 2.4. Pre-Treatment Evaluation

Following the diagnosis of the patient as a case of drug-resistant pulmonary tuberculosis, each patient was subjected to a pretreatment evaluation (clinical and laboratory based). The clinical evaluation included history and physical examinations, and height and weight measurements. Each and every patient was sent to the medicine, ophthalmology, otorhinolaryngology, and cardiology outpatient departments (OPDs) of Darbhanga Medical College and Hospital (DMCH) for clinical check ups. A psychiatric evaluation (if needed) of the patients was conducted by the Department of Psychiatry DMCH. The Pediatric Department and Department of Obstetrics and Gynecology were also involved as per the requirements. Laboratory-based evaluations included plasma glucose; HIV testing; complete blood count; liver function tests; serum T3, T4, and TSH; urine routine examination; serum sodium; serum potassium; serum magnesium; serum calcium; and urinary pregnancy test (in women of a reproductive age group). Laboratory investigations were conducted in the Department of Clinical Pathology, Department of Pathology, and Anti-retroviral treatment (ART) centre, Darbhanga Medical College and Hospital. Chest X-ray/high-resolution computed tomography (HRCT) were conducted in the Radiology Department and an electrocardiogram (ECG) was performed in the Cardiology OPD. The pretreatment evaluations of the patients were conducted to rule out any underlying co-morbid conditions, or radiological, ECG, or bio-chemical derangements.

### 2.5. Treatment Regimens—Indications and Exclusion Criteria

The shorter oral bedaquiline-containing MDR/RR-TB regimen was prescribed for patients of (a) rifampicin-resistant TB (RR-TB); (b) MDR/RR-TB with H resistance (inhA/Kat G mutation only (not both)); and (c) MDR/RR-TB with fluoroquinolone (FQ) resistance not detected. Exclusion criteria included (a) children aged less than 5 years; (b) history of exposure to previous treatment with Bdq, Lfx, Eto, or Cfx for more than one month; (c) extensive pulmonary TB disease; and (d) pregnancy and lactation. The longer oral M/XDR-TB regimen was prescribed for (a) MDR/RR-TB patients who were excluded from the shorter oral bedaquiline-containing MDR/RR-TB regimen, and (b) XDR-TB patients. The replacement of drugs used in the longer oral M/XDR-TB regimen was performed as and when needed (additional resistance, intolerance, unavailability, or contraindication of the drug in the regimen). The most common replacement drugs used were delamanid (during first 6 to 8 months) and ethionamide (during the final 12 months). The H-mono/poly DR-TB regimen was also modified as and when needed, and the replacement drugs used were moxifloxacin and linezolid.

All the drugs used in the DR-TB regimens were administered orally daily under supervision. Adult patients were classified into four weight bands (16–29 kg; 30–45 kg; 46–70 kg; and heavier than 70 kg) for the purpose of dosage of anti-TB drugs. Pediatric patients (under 15 years of age and weighing more than 15 kg) were also classified into four weight bands (16–23 kg; 24–30 kg; 31–34 kg; and heavier than 34 kg) for the same purpose. A dose-drug-administration chart was provided to medical officers and nursing staff at the Nodal DR-TB Centre, Darbhanga. They were instructed to adjust the dose of anti-TB drugs upon an increase in body weight and change in the weight band of the patient. Adverse events were closely monitored and managed.

### 2.6. Follow-Up Monitorings

Treatment follow-up monitoring (clinical, bacteriological, radiological, bio-chemical, and ECG) was conducted at regular intervals at the Darbhanga Medical College and Hospital throughout the course of the DR-TB-treatment-regimen period. During the monitoring of MDR/RR-TB patients on the shorter oral bedaquiline-containing MDR/RR-TB regimen, a sputum culture was performed at the end of month 3, end of month 6, and/or end of treatment. The follow-up evaluation schedule for patients of the longer oral M/XDR-TB regimen included a monthly sputum culture from month 3 onwards to the end of 6, 7, or 8 months based on the previous month’s culture-positive report. Patients on the H-mono/poly DR-TB regimen were monitored by sputum culture performed at the end of month 3, the end of treatment (month 6 and/or 9 if applicable). Follow-up culture results were the basis for declaring the final treatment outcome of all DR-TB patients. All DR-TB patients were clinically evaluated and their weight was measured at monthly intervals. A urinary pregnancy test; complete blood count; Serum T3, T4, and TSH; liver function tests; X-ray chest PA view; and serum electrolytes (sodium, potassium, magnesium, and calcium) were performed as and when clinically indicated. An electrocardiogram (ECG) was conducted at 2 weeks, monthly in first 6 months, and then as and when clinically required.

Post-treatment follow-up monitoring (clinical, radiological, bio-chemical, and ECG) for each treated patient was conducted, with 6 monthly sputum cultures among symptomatic patients, till one year following the completion of the DR-TB-treatment regimen.

### 2.7. Statistics

The data were analyzed and presented as percent, mean, and median. Statistical calculations were conducted by SPSS version 20.0 (SPSS Inc., Chicago, IL, USA).

### 2.8. Definitions

Pulmonary tuberculosis (PTB): A tuberculosis (TB) patient involving lung parenchyma or the tracheo-bronchial tree or both, and who symptomatically presents a cough with mucoid or mucopurulent expectoration for 2 weeks/more.

Extensive pulmonary TB disease: Presence of bilateral cavitary disease or extensive parenchymal damage on chest radiography.

Notified TB case: A pulmonary TB patient who was reported to a case-based web-based real-time patient-management system (https://www.nikshay.in/, accessed on 25 July 2022) of the Government of India and was provided a registration ID (NIKSHAY ID) number.

Rifampicin-resistant TB (RR-TB): A pulmonary TB patient whose bacteriological specimen (sputum) was resistant to rifampicin (R).

Multidrug-resistant TB (MDR-TB): A pulmonary TB patient whose biological specimen (sputum) was resistant to both isoniazid (H) and rifampicin (R).

Extensively drug-resistant TB (XDR-TB): A patient of pulmonary MDR/RR-TB whose biological specimen (sputum) was also resistant to any fluoroquinolone (levofloxacin or moxifloxacin) and at least one additional Group A drug, either bedaquiline or linezolid (or both).

H-mono/poly-drug-resistant-TB: A pulmonary TB patient whose biological specimen (sputum) was isoniazid (H) resistant and rifampicin (R) sensitive. It may have additional resistance to fluoroquinolone (Mfx/Lfx) and/or pyrazinamide (Z).

Cured: A pulmonary TB patient with bacteriologically confirmed TB at the beginning of treatment with evidence of a bacteriological response (bacteriological conversion with no reversion) and no evidence of treatment failed.

Treatment completed: A pulmonary TB patient who completed treatment, but whose treatment outcome did not meet the definitions for cure or treatment failed.

Died: A pulmonary TB patient who died due to any reason during the course of treatment.

Treatment failed: A pulmonary TB patient whose regimen needed to be terminated or permanently changed to a new regimen option or treatment strategy.

Lost to follow-up: A pulmonary TB patient whose treatment was interrupted for two consecutive months or more.

Transferred out: A pulmonary TB patient who was transferred out to another treatment unit and whose treatment outcome was not known and was excluded to lost to follow-up [4].

Grade-I adverse event: Adverse event mild (generally not bothersome).

Grade-II adverse event: Adverse event moderate (bothersome and interfered with activity).

### 2.9. Limitation of the Study

Bio-chemical and hematological data were not correlated with the study outcomes. The susceptibility of isolates for drugs administered was not discussed.

## 3. Results

A total of 268 drug-resistant pulmonary tuberculosis patients with a median age of 27.6 years comprised the study group. Of them, 178 (66.49%) were males and 90 (33.51%) were females with median ages of 28.2 and 26.1 years, respectively. Most of the patients, 162 (60.44%), belonged to the age group of 20–44 years (Table 1 and Table 2).

**Figure 1:**
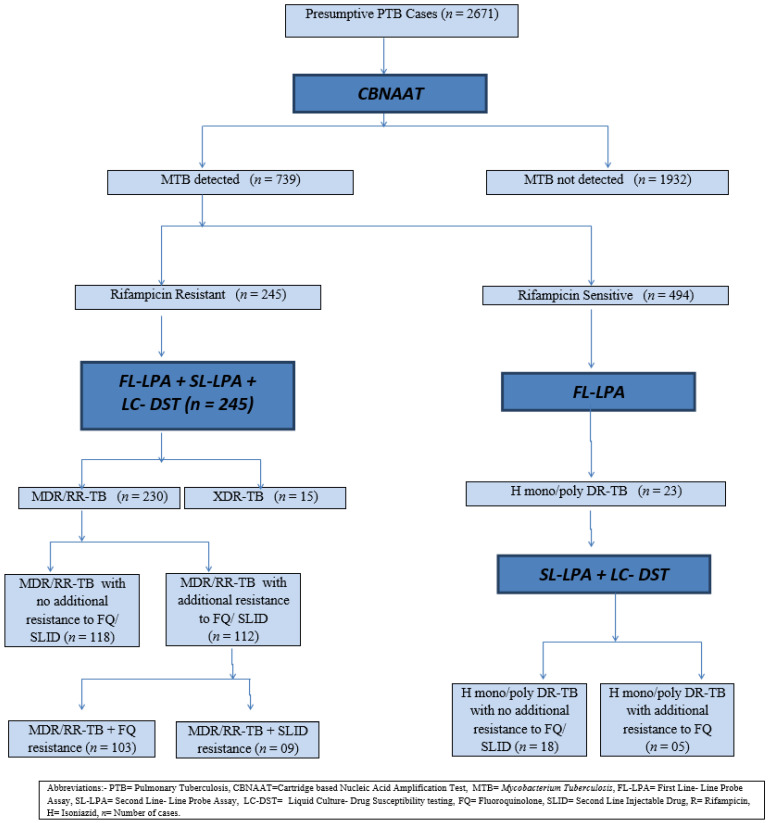
Flowchart—drug-resistant TB diagnosis.

Of all (2671) the presumptive drug-resistant pulmonary tuberculosis cases, 739 (27.64%) cases were observed to be suffering from pulmonary tuberculosis, as *Mycobacterium tuberculosis (MTB)* was detected in the sputum samples obtained by the cartridge-based nucleic acid amplification test (CBNAAT). Out of these, 245 (33.15%) cases were diagnosed by the CBNAAT as rifampicin-resistant tuberculosis (RR-TB), whereas 494 (66.85%) cases presented rifampicin-sensitive tuberculosis (RS-TB) (Figure 1).

All the 245 rifampicin-resistant tuberculosis patients were subjected to a first line- line probe assay, second line-line probe assay, and liquid culture–drug susceptibility tests. MDR/RR-TB was detected in 230 patients and XDR-TB was detected in 15 patients. It was further noted that among the MDR/RR-TB patients, 118 patients had no additional resistance to the fluoroquinolone (FQ) class or second-line injectable-drug (SLID) class; while 112 patients had additional resistance to the FQ/SLID class (103 MDR/RR-TB had additional resistance to the FQ class and 09 MDR/RR-TB with additional resistance to the SLID class).

All the 494 rifampicin-sensitive tuberculosis patients were subjected to the first line-line probe assay. H-mono/poly-drug-resistant tuberculosis was detected among 23 patients. These 23 patients were further subjected to a second line-line probe assay and liquid culture–drug susceptibility testing, which revealed that, out of these, 18 patients had H-mono/poly-drug-resistant tuberculosis with no additional resistance to the fluoroquinolone class (FQ) or second-line injectable-drug (SLID) class, while 05 patients had H-mono/poly-drug-resistant tuberculosis with additional resistance to the fluoroquinolone (FQ) class.

A total of 41.30%, 13.33%, and 13.04% of MDR/RR-TB, XDR-TB, and H-mono/poly-DR-TB patients, respectively, had treatment outcomes declared as cured. Thus, a higher cure rate was reported for MDR/RR-TB patients than XDR-TB and H-mono/poly-DR-TB patients. It was also noted that 10.43%, 13.33%, and 73.91% of MDR/RR-TB, XDR-TB, and H-mono/poly-DR-TB patients, respectively, were declared as having treatment completed. Thus, more treatment outcomes as treatment completed were recorded in H-mono/poly-DR-TB patients. A greater incidence of death was observed in XDR-TB patients in comparison to MDR/RR-TB patients (60.0% vs. 20.43%). No death and treatment failure was reported for H-mono/poly-DR-TB patients (Table 3).

Many adverse events (AEs) were observed during the total duration of the DR-TB-treatment regimen. A total of 193 (72. 01%) patients had grade I and 75 (27.98%) had grade II AEs. Gastrointestinal symptoms were the most common of all. QTcF (corrected-QT interval Fridericia) prolongation was observed in some cases. The investigations conducted during the course of treatment revealed that serum aspartate aminotransferase/serum alanine transaminase was elevated higher than the upper level of normal in 20 (7.46%) patients and serum uric acid was elevated to higher than 7.0 mg/dL in 10 (3.37%) patients (Table 4).

All characteristics observed as significant are shown in Table 5.

A total of 15 patients of drug-resistant pulmonary TB were observed to be co-infected with human immunodeficiency virus (HIV) infection, out of which 14 (93.33%) had MDR/RR-TB and 1 (6.66%) had XDR-TB. The treatment outcomes of MDR/RR-TB patients revealed that 7 were cured (50%), 2 treatment completed (14.28%), 4 died (28.57%), 1 and failed (7.24%). The treatment outcome of an XDR-TB patient co-infected with HIV was 1 died (100%).

## 4. Discussion

The present study reported on the predominance of the young age group and males among drug-resistant pulmonary tuberculosis cases. This result is in concordance with many previous studies. The age group and gender predominance in DR-TB may be related to the mobility and activity (greater exposure) levels of the population in particular zones [5,6,7,8,9].

The present study reported the successful treatment outcomes (sum of cured and treatment-completed patients) in 119 (51.73%), 4 (26.66%), and 20 (86.95%) cases in MDR/RR-TB, XDR-TB, and H-mono/poly-DR-TB patients, respectively; while unsuccessful treatment outcomes (died, treatment failed, lost to follow-up, and transferred out) presented in 111 (58.26%), 11 (73.33%), and 3 (13.04%) cases in MDR/RR-TB, XDR-TB, and H-mono/poly-DR-TB patients, respectively. Several studies conducted worldwide revealed successful treatment outcomes ranging from 43.50% to 80.40% in MDR/RR-TB and XDR-TB patients treated with bedaquiline-containing regimens. This variability in successful treatment outcomes may be due to the different demographic profiles of patients and their co-morbidities [10,11,12,13,14,15,16,17,18].

The factors associated with treatment outcomes as failing in patients with drug-resistant pulmonary TB were low-body mass index, HIV-DR-TB co-infection, diabetes DR-TB co-infection, bilateral cavities/extensive parenchymal disease on X-ray chest/HRCT chest, poor tolerability of anti-TB drugs, and additional resistance developing during the course of a treatment regimen.

Adverse events (AEs) were reported in most of the 199 (74.25%) drug-resistant pulmonary tuberculosis patients. A total of 97 (36.19%) cases presented more than one adverse event. The most common adverse events were gastrointestinal and they were related to the total number of tablets/capsules taken orally by the patients per day during the treatment period, as well as offending drugs. The next common AE was peripheral neuropathy related to linezolid. Patients complained of pain in the lower limb/limbs. A neurologist’s opinion was taken and these patients were managed with supportive drugs and a tapering of doses of linezolid to 300 mg after the initial 6–8 months of treatment. In three adult MDR/RR-TB patients, QTcF prolongation of more than 500 ms was observed within two weeks upon the initiation of the treatment regimen; Bdq administration was ended and replaced with delamanid.

Arthralgia (1.17% to 37%), gastrointestinal symptoms (6.2% to 41%), peripheral neuropathy (2.5% to 23.3%), visual disturbance (1.1% to 2.5%), psychiatric changes (3.4% to 5.0%), electrolyte disturbance (4.8% to 17.5%), and QTcF prolongation of less than 500 ms (4.90% to 31.60%) have been reported by several studies in patients on the MDR/RR-TB on bedaquiline-containing DR-TB-treatment regimen [10,11,12,13,14,15,16,17,18,19,20].

The present study observed a higher incidence of some AEs (gastrointestinal, psychiatric, and arthralgia) and a lower incidence of QTcF prolongation (to less than 500 ms) in comparison to the other studies. All of these AEs were well managed by consultants (members of the DR-TB Core Committee) from different departments of the Darbhanga Medical College and Hospital.

The present study did not observe hematological abnormalities reported to be associated with linezolid (leucopenia, thrombocytopenia, anemia, red cell aplasia, coagulation abnormalities, and eosinophilia); ethionamide (hypothyroidism and hyperglycemia); amikacin (severe hypokalemia, nephrotoxicity, and hearing loss); fluoroquinolone (tendonitis and tendon rupture); cycloserine (seizures); linezolid and ethambutol (optic atrophy), and anaphylaxis, as mentioned in some reports. No drug-related mortality occurred in our cohort study. Linezolid-associated adverse events reported by others were probably related to dose and duration (600 mg per day for 13 months). We used linezolid at a dose of 600 mg per day (for patients in the weight bands of 30–45 kg; 46–70 kg, and heavier than 70 kg) for the initial 6 months, and then the dose of linezolid was tapered to 300 mg per day. For patients weighing less than 30 kg, linezolid was used at a dose of 300 mg per day from the start of the treatment [21,22].

We expect an increase in the rate of successful treatment outcomes in the future. The use of new drugs will make us drugs friendly. The deaths that occurred in the present study were associated with extensive TB disease, HIV-DR-TB co-infection, and diabetes-DR-TB co-infection. Loss to follow-up may be related to socio-economic causes and should be addressed. Cardiological adverse events were not a matter of concern in the present study, and this satisfied us.

## 5. Conclusions

Gender, literacy, co-infection, diabetes mellitus, alcohol intake, smoking habit, low body mass index and poverty have a significant impact on the treatment outcome of drug-resistant pulmonary tuberculosis patients. Interventions are needed to reduce the number of treatment failures, deaths, loss of follow-ups and transferred out cases. We recommend strengthening of the follow-up monitoring system, timely detection and management of associated co-infection, increased nutritional and economic support to the poor patients for more successful treatment outcomes of drug resistant pulmonary tuberculosis patients.

## Figures and Tables

**Table 1:** Age and gender distribution of drug-resistant pulmonary tuberculosis patients (*n* = 268).

Age Group (in Years)	Male *n* (%)	Female *n* (%)	Total *n* (%)
10–14	1 (0.37)	5 (1.87)	6 (2.24)
15–19	25 (9.33)	19 (7.08)	44 (16.42)
20–24	47 (17.53)	18 (6.71)	65 (24.25)
25–29	25 (9.33)	11 (4.10)	36 (13.43)
30–34	15 (5.59)	15 (5.59)	30 (11.19)
35–39	6 (2.23)	6 (2.23)	12 (4.48)
40–44	17 (6.34)	2 (0.75)	19 (7.08)
45–49	11 (4.10)	3 (1.11)	14 (5.22)
50–54	8 (2.99)	4 (1.49)	12 (4.48)
55–59	5 (1.87)	-	5 (1.87)
60–64	9 (3.36)	3 (1.11)	12 (4.48)
65–69	6 (2.24)	1 (0.37)	7 (2.61)
70–74	3 (1.11)	3 (1.11)	6 (2.24)
Total	178 (66.49)	90 (33.51)	268 (100)

Abbreviations: *n* = number of cases, % = percent.

**Table 2:** Mean and median ages of drug-resistant tuberculosis patients.

Group	Mean Age (Years)	Median Age (Years)
Male patients (*n* = 118)	33.5	28.2
Female patients (*n* = 90)	29.7	26.1
Total patients (*n* = 268)	32.3	27.6

Abbreviations: *n* = number of cases, % = percent.

**Table 3:** Final treatment outcome of drug-resistant pulmonary tuberculosis patients (*n* = 268).

	MDR/RR-TB (*n* = 230)	XDR-TB (*n* = 15)	H-mono/poly DR-TB (*n* = 23)	Total DR-TB Patients (*n* = 268)
	*n* (%)	*n* (%)	*n* (%)	*n* (%)
Cured	95 (41.30)	02 (13.33)	03(13.04)	100 (37.31)
Treatment completed	24 (10.43)	02 (13.33)	17 (73.91)	43 (16.04)
Died	47 (20.43)	09 (60.00)	00	56 (20.89)
Treatment failed	22 (9.57)	00	00	22 (8.21)
Lost to follow up	31 (13.48)	02 (13.33)	01 (4.35)	34 (12.69)
Transferred out	11 (4.78)	00	02 (8.69)	13 (4.85)

Abbreviations: RR = rifampicin-resistant tuberculosis, MDR-TB = multi-drug-resistant tuberculosis, XDR-TB = extensively drug-resistant tuberculosis, H-isoniazid DR-TB = drug-resistant tuberculosis, *n*= number of cases, % = percent.

**Table 4:** Frequency of adverse events (AEs) among drug-resistant pulmonary tuberculosis patients (*n* = 268).

Grouped AEs	Specific AEs	Frequency of AEs *n* (%)	Offending Anti-TB Drug
Gastrointestinal	Nausea	11 (4.10)	Pyrazinamide, ethionamide
	Vomiting	10 (3.73)	Pyrazinamide, ethionamide
	Fullness of abdomen	119 (44.40)	Pyrazinamide, ethionamide
	Pain abdomen	70 (26.12)	Pyrazinamide, ethionamide
Neurological	Peripheral neuropathy	61 (22.76))	Linezolid
Psychiatric	Psychosis	37 (13.80)	Cycloserine
	Depression	32 (11.94))	Cycloserine
Skeletal	Arthralgia	39 (14.55)	Pyrazinamide
Ophthalmic	Visual disturbance	5 (1.86)	Linezolid, ethambutol
Endocrinal	Hypothyroidism	2 (0.74)	Ethionamide
Dermatological	Skin discoloration	21 (7.83)	Clofazimine
Cardiological	QTcF prolongation	31(12.60)	Bedaquiline

Abbreviations: AEs = adverse events, *n* = number of cases, % = percent, QTcF = QT interval corrected using Fridericia’s formula.

**Table 5:** Associations between background characteristics and treatment outcomes (*n* = 268).

Characteristics	Values	Treatment Unsuccessful	Treatment Successful	*p*-Value
		*n* = 125	*n* = 143	
Age in years (mean)	32.3	39.8	25.6	0.000
Gender	Male	96 (76.8%)	82 (57.3%)	0.008
	Female	29 (23.2%)	61 (47.2%)	
Education	Illiterate	41 (32.8%)	21 (14.7%)	0.005
	Literate and above	84 (67.2%)	122 (85.3%)	
Income	Below poverty line	35 (28%)	13 (9.1%)	0.001
	Above poverty line	90 (72)	130 (90.9%)	
Diabetes	Yes	19 (15.2%)	10 (7%)	0.031
	No	106 (84.8%)	133 (93%)	
Body mass index (BMI)	Low (<18.5)	39 (31.2%)	21 (14.7%)	0.0012
	Normal	86 (68.8%)	122 (85.3%)	
Alcohol	Yes	14 (11.2%)	4 (2.8%)	0.0061
	No	111 (88.8%)	139 (97.2%)	
Tobacco use	Yes	31 (24.8%)	19 (13.3%)	0.0158
	No	94 (75.2%)	124 (86.7%)	

Successful (cured + treatment completed); unsuccessful (died + failed + lost to follow up + transferred out).

## Data Availability

The data that support the findings of this study are available from the corresponding author upon reasonable request.

## References

[1] (2021). Global Tuberculosis Report 2021.

[2] WHO Global Lists of High Burden Countries for Tuberculosis (TB), TB/HIV and Multidrug/Rifampicin-Resistant TB (MDR/RR-TB), 2021–2025: Background Document. https://apps.who.int/iris/handle/10665/341980.

[3] (2021). Impact of the COVID-19 Pandemic on TB Detection and Mortality in 2020. World Health Organization. https://www.who.int/publications/m/item/impact-of-the-covid-19-pandemic-on-tb-detection-and-mortality-in-2020.

[4] Guidelines on Programmatic Management of Drug—Resistant Tuberculosis in India 2021. Central TB Division, Directorate General of Health Services, Ministry of Health & Family Welfare, Government of India, New Delhi. https://tbcindia.gov.in/showfile.php?lid=3590.

[5] India TB Report 2022. Central TB Division, Directorate General of Health Services, Ministry of Health & Family Welfare, New Delhi. https://tbcindia.gov.in/index1.php?lang=1&level=1&sublinkid=5613&lid=3658.

[6] Molecular Assays Intended as Initial Tests for the Diagnosis of Pulmonary and Extrapulmonary Tuberculosis and Rifampicin Resistance in Adults and Children: Policy Update. World Health Organization: Geneva, Switzerland, 2020. https://apps.who.int/iris/handle/10665/330395.

[7] Waghmare M., Ketaki U., Desai U., Joshi J. (2017). Drug resistant tuberculosis at a drug resistant tuberulosiscentre, India-Analysis of outcome. Eur. Respir. J..

[8] Schnippel K., Ndjeka N., Maartens G., Meintjes G., Master I., Ismail N., Hughes J., Ferreira H., Padanilam X., Romero R. (2018). Effect of bedaquiline on mortality in South African patients with drug-resistant tuberculosis: A retrospective cohort study. Lancet Respir. Med..

[9] Borisov S.E., Dheda K., Enwerem M., Romero Leyet R., D’Ambrosio L., Centis R., Sotgiu G., Tiberi S., Alffenaar J.W., Maryandyshev A. (2017). Effectiveness and safety of bedaquiline-containing regimens in the treatment of MDR- and XDR-TB: A multicentre study. Eur. Respir. J..

[10] Lofranco V.S., Torres-Cervas M.R.A., Asence K.A., Del Mundo K.M.A., Balanag V.M., Santiago M.R.T., Garfin A.M.C.G. (2022). Interim Outcomes and Adverse Events among Drug-Resistant Tuberculosis Patients Treated with Bedaquiline in the Philippines. J. Tuberc. Res..

[11] Gao M., Gao J., Xie L., Wu G., Chen W., Chen Y., Li L. (2021). Early outcome and safety of bedaquiline-containing regimens for treatment of MDR-and XDR-TB in China: A multicentre study. Clin. Microbiol. Infect..

[12] Sharma P., Lalwani J., Pandey P., Thakur A. (2019). Factors associated with the development of secondary multi-resistant tuberculosis. Int. J. Prev. Med..

[13] Mullerpattan J.B., Udwalia Z.Z., Banka R.A., Ganatra S.R., Udwalia Z.F. (2019). Catastrophic costs of treating drug resistant TB patients in a tertiary care hospital in India. Indian J. Tuberc..

[14] Soeroto A.Y., Pratiwi C., Santoso P., Lestari B.W. (2021). Factors affecting outcome of longer regimen multidrug-resistant tuberculosis treatment in West Java Indonesia: A retrospective cohort study. PLoS ONE.

[15] Putra O.N., Hidayatullah A.Y. (2021). Treatment outcomes of patients with multidrug-resistant tuberculosis: Concern to bedaquiline. Tuberc. Respir. Dis..

[16] Zhao Y., Fox T., Manning K., Stewart A., Tiffin N., Khomo N., Wasserman S. (2019). Improved treatment outcomes with bedaquiline when substituted for second-line injectable agents in multidrug-resistant tuberculosis: A retrospective cohort study. Clin. Infect. Dis..

[17] Kang H., Jo K.W., Jeon D., Yim J.J., Shim T.S. (2020). Interim treatment outcomes in multidrug-resistant tuberculosis using bedaquiline and/or delamanid in South Korea. Respir. Med..

[18] Chesov D., Heyckendorf J., Alexandru S., Donica A., Chesov E., Reimann M., Crudu V., Botnaru V., Lange C. (2021). Impact of bedaquiline on treatment outcomes of multidrug-resistant tuberculosis in a high-burden country. Eur. Respir. J..

[19] Mbuagbaw L., Guglielmetti L., Hewison C., Bakare N., Bastard M., Caumes E., Fréchet-Jachym M., Robert J., Veziris N., Khachatryan N. (2019). Outcomes of Bedaquiline Treatment in Patients with Multidrug-Resistant Tuberculosis. Emerg. Infect. Dis..

[20] Ndjeka N., Campbell J.R., Meintjes G., Maartens G., Schaaf H.S., Hughes J., Padanilam X., Reuter A., Romero R., Ismail F. (2022). Treatment outcomes 24 months after initiating short, all-oral bedaquiline-containing or injectable-containing rifampicin-resistant tuberculosis treatment regimens in South Africa: A retrospective cohort study. Lancet Infect. Dis..

[21] Jaspard M., Butel N., El Helali N., Marigot-Outtandy D., Guillot H., Peytavin G., Veziris N., Bodaghi B. (2020). Linezolid-Associated Neurologic Adverse Events in Patients with Multidrug-Resistant Tuberculosis, France. Emerg. Infect. Dis..

[22] Pratama N.Y., Zulkarnain B.S., Soedarsono S., Fatmawati U. (2021). Hematological side effect analysis of linezolid in MDR-TB patients with individual therapy. J. Basic Clin. Physiol. Pharmacol..

